# Advances of Regulatory B Cells in Autoimmune Diseases

**DOI:** 10.3389/fimmu.2021.592914

**Published:** 2021-04-15

**Authors:** Qiugang Zhu, Ke Rui, Shengjun Wang, Jie Tian

**Affiliations:** ^1^ Department of Immunology, Jiangsu Key Laboratory of Laboratory Medicine, School of Medicine, Jiangsu University, Zhenjiang, China; ^2^ Department of Laboratory Medicine, Affiliated Hospital of Jiangsu University, Zhenjiang, China

**Keywords:** B cells, regulatory B cells, IL-10^+^ B cells, immunomodulation, autoimmune diseases

## Abstract

With the ability to induce T cell activation and elicit humoral responses, B cells are generally considered as effectors of the immune system. However, the emergence of regulatory B cells (Bregs) has given new insight into the role of B cells in immune responses. Bregs exhibit immunosuppressive functions *via* diverse mechanisms, including the secretion of anti-inflammatory cytokines and direct cell contact. The balance between Bregs and effector B cells is important for the immune tolerance. In this review, we focus on recent advances in the characteristics of Bregs and their functional roles in autoimmunity.

## Introduction

Regulatory B cells (Bregs) are immunosuppressive cells that downregulate immune responses and support immunological tolerance (1). The roles of Bregs in AIDs have been widely reported, such as type 1 diabetes (T1D), rheumatoid arthritis (RA), multiple sclerosis (MS), systemic lupus erythematosus (SLE), and inflammatory bowel disease (IBD) (2–5). Moreover, Bregs could be biomarkers of treatment responses, including methotrexate and rituximab treatments (6, 7).

Unlike natural Tregs, specific transcriptional factors of Bregs have not been discovered because of the diversity of suppressive mechanisms and signals for induction. The identification of Bregs is dependent on their immunomodulatory effects, such as inhibition of T cell activation and cytokine secretion (1, 8). Evidence that B cells could regulate immune responses was firstly demonstrated in 1974, and progress has been made in the past few decades, including the phenotypes, functional molecules, *in vitro* induction, as well as expanding number of diseases implicated (9). There are three models that explain the generation of Bregs: (1) multi-lineage Bregs, suggesting that subsets of Bregs can generate from B cells at different stages, such as IL-10^+^ B cells; (2) single-lineage Bregs, meaning that subsets of Bregs derive from a specific progenitor (B1 or B2 cells), such as CD5^+^CD1d^+^ B cells; and (3) induced Bregs, indicating that Bregs can differentiate from any B cell upon stimulation with specific stimuli, such as BAFF or IL-1β/IL-6-induced IL-10^+^ B cells (3, 10–13). The suppresssive activities of Bregs are mainly related to the secretion of anti-inflammatory cytokines (by IL-10, IL-35, etc.) and/or the expression of inhibitory molecules (PD-1/PD-L1 and FasL). In this review, we focus on recent advances in the characteristics of Bregs and their functions in AIDs.

## Characteristics of Bregs

The suppressive activities of Bregs and the molecules that carry out their suppressive functions have been partially described, including the inhibition of T cell activation, induction of Tregs, the expression of IL-10, IL-35, TGF-β, and PD-1/PD-L1. Some specific markers used for the identification of Bregs have been elucidated, but there are some phenotypic overlaps among Breg subsets (**Table 1**).

**Table 1:** Phenotype of Breg subsets in mouse and human.

Species	Subtype	Phenotype	Functional molecules	References
Mouse	B10	CD19^+^CD5^+^CD1d^hi^	IL-10	(11, 14)
	MZ B	IgM^hi^IgD^lo^CD21^hi^CD23^−^CD1d^hi^	IL-10	(15)
	B-1a	CD90^−^CD5^+^	IL-10	(16)
	T2-MZP	B220^+^CD21^hi^CD23^+^IgM^hi^ CD1d^hi^	IL-10	(17)
	Plasma	CD19^+^CD138^+^IgM^+^	IL-10, IL-35	(18)
	Plasmablasts	CD138^+^CD44^hi^	IL-10	(19)
	Tim-1^+^ B	CD19^+^Tim-1^+^	IL-10	(20)
	i35-Breg	CD5^+^CD1d^hi^FcγIIb^hi^	IL-35	(21)
	GITRL^+^ B	–	GITRL	(22)
	Killer B	CD19^+^CD5^+^FasL^+^	FasL, TGF-β	(23, 24)
	PD-L1^hi^ B	CD19^+^PD-L1^hi^	PD-L1	(25)
	GIFT-15 B	B220^+^CD21^+^CD22^+^CD23^+^ CD24^+^CD1d^+^CD138^+^IgD^+^IgM^+^	IL-10	(26)
	–	B220^+^CD39^+^CD73^+^	Adenosine, CD39^+^CD73^+^ EVs	(27, 28)
Human	transitional B	CD19^+^CD24^hi^CD38^hi^	IL-10	(4)
	Memory B	CD19^+^CD24^hi^CD27^+^	IL-10	(29)
	Br1	CD25^hi^CD71^hi^CD73^lo^	IL-10	(30)
	TIM1^+^ B	CD19^+^TIM1^+^	IL-10	(31)
	Plasmablasts	CD19^lo^CD27^hi^CD38^hi^	IL-10	(32, 33)
	IgA^+^ B	CD19^+^IgA^+^	IL-10, PD-L1	(34)
	Exhausted B	CD19^+^CD95^+^	CD95	(35)
	Killer B	CD19^+^CD38^+^IgM^+^FasL^+^	FasL	(36)
	PD-L1^+^ B	CD19^+^PD-L1^+^	PD-L1	(25)
	–	CD19^+^CD39^+^	Adenosine	(37)
	iBreg	–	TGF-β, IDO	(38)
	Others	CD19^+^FoxP3^+^,CD19^+^TGFβ^+^	TGF-β	(39)

IL-10-producing B cells are regulatory B cell subsets with the capacity to downregulate immune responses *via* IL-10. IL-10 production is the most studied suppressive mechanism that most investigated, there are some phenotypes within IL-10^+^ B cells. Several studies have confirmed that human CD19^+^CD24^hi^CD38^hi^ B cells, a phenotype that has been related to transitional B cells, comprise the highest fraction of IL-10^+^ B cells in human peripheral blood upon stimulation with CD40L, CpG, Brefeldin A, phorbol 12-myristate 13-acetate and ionomycin (2, 40). Similar to human transitional B cells, mouse CD19^+^CD21^hi^CD24^hi^CD23^hi^ transitional 2 marginal zone precursor (T2-MZP) B cells are also capable of producing IL-10. The inhibitory function of T2-MZP B cells depends on IL-10 production because anti-IL-10 or anti-IL-10R treatments eliminate the inhibitory effect of B cells on IFN-γ secretion by CD4^+^ T cells (17). Human CD24^+^CD27^+^ B cells, a phenotype reminiscent of memory B cells, also have been characterized as the major source of IL-10^+^ B cells upon stimulation with CpG and CD40L (3). Also, CD24^low/neg^CD38^hi^ plasmablast-like regulatory B cells are the members of IL-10^+^ B cells in human (41). Mouse CD138^+^CD44^hi^ plasmablasts and plasma cells cell-derived IL-10 inhibited the generation of CD4^+^IFN-γ^+^ and CD4^+^IL-17^+^ T cells (19). In mice, CD1d^hi^CD5^+^ B cells are the main subsets of IL-10^+^ B cells. Adoptive transfer of CD1d^hi^CD5^+^ B cells to mice could prevent experimental autoimmune myasthenia gravis associated with downregulation of mature dendritic cell markers and expansion of Tregs (42). CD21^hi^CD23^−^ marginal zone (MZ) B cells were producers of IL-10 upon stimulation with inflammatory stimuli including TLR9 and TLR4, and adoptive cell transfer experiments in which the absence of IL-10-producing B cells conferred the host a greater capability to induce Th1 responses and clear the infection (15, 43, 44). Besides, mouse B-1 cells, Tim-1^+^ B cells in both human and mice have been revealed to exert their functions in an IL-10-dependent manner (16, 20, 31).

In addition to IL-10-producing B cells, Breg subsets function through other mechanisms also have been widely reported. PD-L1^+^ B cells limited the expansion of human Tfh cells and the proliferation of mouse CD8^+^ T cells, these cells functioned through the interaction between PD-1 and PD-L1 (25, 45). FasL is expressed on both human and mouse B cells, these B cells are termed as killer B cells and have been confirmed to induce immune tolerance *via* FasL (36, 46, 47). As a receptor of FasL, CD95 (also called Fas) is expressed on CD24^high^CD38^high^ and CD5^+^ B cells, these B cells are termed as CD95^+^ exhausted Bregs and are positively associated with severe colitis in human (35). Interleukin-35 was a novel anti-inflammatory cytokine of the IL-12 family cytokines and was found to be produced by human B cells and mouse CD138^+^ plasma cells (48, 49). IL-35 was shown to induce the expression of itself by B cells, as well as IL-10 (48, 50). Adenosine-producing B cells are also the subsets of Bregs, including human CD19^+^CD39^+^ B cells and murine B220^+^CD39^+^CD73^+^ cells, and CD39 and CD73 hydrolyze ATP to produce adenosine (27, 51). Also, TGF-β-producing B cells are non-negligible subgroups in Bregs, inhibiting T cell proliferation and inducing the generation of Tregs (38).

As described above, we can find that Bregs contain diverse subsets, and IL-10^+^ B cells are the major subsets. Most Bregs exert their functions *via* producing anti-inflammatory cytokines and expressing inhibitory molecules, and the importance of these cytokines and inhibitory molecules has been evidenced with experiments *in vivo* and *in vitro*.

## Functions of Bregs in autoimmune diseases

B cells are critical members of humoral immunity with the ability to produce autoantibodies and to present antigens, traditionally thought to play a pathogenic role in AIDs. However, numerous studies have characterized the immunoregulatory functions of Bregs in AIDs (52). Here, Bregs have been reported to exert inhibitory effects through IL-10, IL-35, TGF-β, and PD-1/PD-L1 in both patients with AIDs and murine models. In the following sections, we will discuss the role of Bregs in inflammatory bowel disease (IBD), systemic lupus erythematosus (SLE), rheumatoid arthritis (RA), primary sjögren’s syndrome (pSS), type 1 diabetes (T1D), thyroid autoimmune disorders, multiple sclerosis (MS), and other AIDs.

### Bregs in Inflammatory Bowel Disease

Inflammatory bowel disease is a chronic inflammatory disease, including ulcerative colitis (UC) and Crohn’s disease (CD). The etiology of IBD is related to microbiota, genetics, environmental factors, and imblanced Th17/Treg responses (53, 54). Transfer of microbiotas from IBD patients into germfree mice increased numbers of intestinal Th17 cells and Th2 cells and decreased numbers of RORγt^+^ Tregs, indicating a pathogenic role of Th cells caused by microbiotas (55). In patients with CD, the ability of B cells to produce IL-10 was impaired, and the frequency of CD19^+^CD1d^+^IL-10^+^ B cells was decreased in PBMCs. In this study, the presence of CD was related to the decreased production of IL-10 by peripheral blood B cells (5). Patients with UC also exhibited decreased frequencies of CD24^hi^CD38^hi^ and CD5^+^ Bregs in the peripheral blood and intestinal tissues. Besides, mayo clinic scores, C-reactive protein (CRP), and erythrocyte sedimentation rate (ESR) in UC patients were negatively correlated with the frequency of Bregs in PBMCs (35). In patients with UC, serum vasoactive intestinal peptide (VIP) levels were positively correlated with IL-10 mRNA expression in CD19^+^CD73^−^CD25^+^CD71^+^ Bregs, indicating that VIP might regulate the function of Bregs and the further exploration has confirmed it in murine models (56). Suppressive role of B cells in chronic colitis were demonstrated by Mizoguchi et al., they found that B cells and Igs could suppress colitis induced by the transfer of mesenteric lymph node (MLN) cells from TCR-α^−/−^ × Igμ^−/−^ mice, presumably by affecting the clearance of apoptotic cells (57). A later study conducted by Mizoguchi et al. revealed that CD1d-expressing B cells suppressed the progression of intestinal inflammation, which was associated with the enhanced IL-10 expression in MLN CD1d^+^ B cells (58). Dextran sulfate sodium (DSS) induced chronic colitis is a murine model of human CD. In this model, Wang et al. reported that B cells could suppress DSS-induced colitis in an IL-10 independent manner because an adoptive transfer of *Il-10^−/−^* B cells also attenuated colitis. In this study, B cells contributed to the maintenance of gut-associated lymphoid tissues (GALT) Tregs that in turn promoted B-cell differentiation into IgA-producing plasma cells, then prevented excessive immune responses that can lead to colitis (59). IL-33, IL-35, bacterial immunogenicity, and endometrial regenerative cells have been revealed to maintain/expand Bregs and ameliorate colitis, indicating a protective role of Bregs in IBD (60–64).

### Bregs in Systemic Lupus Erythematosus

Systemic lupus erythematosus (SLE) is an autoimmune inflammatory disease that occurs more frequently in females and is characterized by the breakdown of immune tolerance, high levels of autoantibody production, and multiple organ damage (65). Patients with SLE exhibit deficiencies in the function of Bregs. CD19^+^CD24^hi^CD38^hi^ B cells isolated from healthy individuals exerted regulatory capacity, but these cells derived from SLE patients lost the ability to inhibit the expression of IFN-γ and TNF-α by CD4^+^ T cells (4). Heinemann and colleagues identified that the percentage of CD19^+^CD24^hi^CD38^hi^ B cells in SLE patients was similar to that of healthy individuals (66). In contrast, another report described an increase of CD19^+^CD24^hi^CD38^hi^ B cells in patients with SLE (67). Such discrepancies may be attributed to the complexity of the disease, different stages of diseases, and physiological environments within individuals. Plasmacytoid dendritic cells (pDCs) promoted the differentiation of immature B cells into Bregs *via* IFN-α and CD40-CD40L in healthy individuals. This form of immune tolerance was deficient in SLE patients because pDCs derived from SLE patients promoted plasmablast differentiation by producing relatively higher levels of IFN-α. Notably, newly repopulated immature B cells in SLE patients responding to rituximab showed normalized expression of STAT1 and STAT3 and could differentiate into CD24^+^CD38^hi^ Bregs (68). The iNKT cell number and function were rescued in SLE patients responding to rituximab upon normalization of CD1d expression in repopulated immature B cells, indicating an important role of immature B cells in mataining the homeostasis of iNKT cells (69). MRL-Fas^lpr/lpr^ mice and NZB/NZW (NZB/W) F1 mice are murine models used to investigate SLE. BAFF is a cytokine generally thought to be crucial to B cell maturation and survival, it could induce IL-35 production by CD5^+^CD1d^hi^FcγRIIb^hi^ Bregs in MRL-Fas^lpr/lpr^ mice. These IL-35-producing Bregs suppressed inflammatory cytokines (including TNF-α and IFN-γ) production by conventional CD4^+^ T cells and promoted the expansion of Tregs (21). Intriguingly, treatment of mice with IL-35 enriched IL-10^+^ Bregs in mild and moderate SLE mice as well as peripheral blood cells in severe SLE mice, which was accompanied by the expansion of Tregs (70). Taken together, the microenvironment within SLE patients could likely interfere with the generation of Bregs and impair their functions. Moreover, recovering the function of Bregs may be a method to ameliorate SLE.

### Bregs in Rheumatoid Arthritis

Rheumatoid arthritis (RA) is a chronic inflammatory autoimmune disease characterized by synovial hyperplasia and bone destruction of the joints, affecting about 0.5–1.0% of adults in developed countries (71). Collagen-induced arthritis (CIA) is induced by immunization bovine or chicken collagen in a susceptible strain of DBA/1 mice or C57/BL6 mice, and CIA mice have become useful animal models of RA (72, 73). The involvement of B cells in RA has been well recognized, such as the precence of anti-citrullinated protein antibodies (ACPAs), rheumatoid factor (RF), higher total serum IgA, and elevated level of unmutated IgG^+^ B cells compared to healthy controls (74, 75). As important immune regulators, Bregs have been revealed in RA patients with impaired functions (76). In addition, IL-10^+^ B cells were inversely related to DAS28 and the levels of RF (77). As members of Bregs, CD24^hi^CD38^hi^ and CD24^hi^CD27^+^ B cells from RA patients lost the ability to convert CD4^+^CD25^−^ T cells into regulatory T cells (77). Furthermore, circulating CD19^+^CD24^hi^CD38^hi^ Bregs are biomarkers of response to methotrexate in early rheumatoid arthritis patients (6). Besides, the frequency of CD19^+^CD24^hi^CD27^+^ regulatory B10 cells was increased in patients treated with a TNF inhibitor (78). PD-L1^+^ Bregs with CD8^+^ T cell suppressive capacity activity were decreased in untreated RA patients compared to healthy dornors. With successful treatment (methotrexate, TNF inhibitors, or JAK inhibitors), PD-L1^+^ B cells were increased in patients (45). Similarly, several Bregs inhibit the development of the CIA have been reported. Evans et al. found that T2-MZP B cells suppressed arthritis through the inhibition of type II collagen (C II) specific T cell activation and Th1 response. This process was dependent on IL-10 because T2-MZP B cells purified from IL-10 knockout mice failed to alleviate arthritis (17). Also, CD1d^+^ T2-MZP Bregs induced suppressive invariant natural killer (iNKT) cells *via* CD1d-lipid presentation, then secreted IFN-γ by iNKT cells resulted in the downregulation of Th1 and Th17 immune responses and amelioration of antigen-induced arthritis (79). Yang and colleagues reported that BAFF-induced IL-10-producing CD5^+^CD1d^hi^ B10 cells inhibited the proliferation of naïve T cells, accompanied by decreased expression of RORγt (a key transcriptional factor for Th17 cells) and the differentiation of Th17 cells, consequently resulted in the amelioration of CIA (13, 80). Besides, FoxP3-expressing B cells ameliorated autoimmune arthritis *via* regulating the balance of Treg/Th17 cells (81). Thus, Bregs inhibit the development of RA through the reduction of Th responses and enhanced Tregs responses, and expansion of Bregs *in vitro* can be a promising treatment of arthritis.

### Bregs in Thyroid Autoimmune Disorders

Graves’ disease and Hashimoto’s thyroiditis (HT) are two common thyroid autoimmune disorders (AITD), characterized by the presence of circulating anti-thyroid antibodies (including pathognomonic activating autoantibodies, autoantibodies to the thyroid self-antigens thyroglobulin and thyroid peroxidase), and lymphocytic infiltration into the thyroid. T cells, B cells, and DCs are actively involved in the pathogenesis of diseases (82). A study of both types of patients (Graves’ disease and HT) showed a tendency for decreased numbers of CD19^+^CD24^+^CD27^+^IL-10^+^ and CD19^+^IL-10^+^ B cells, which could be responsible for immune imbalance and AITDs (83). Co-occurrence of AIDs within a patient is a common phenomenon in rheumatic diseases, AITD are also the most frequent diseases associated with polyautoimmunity (84, 85). HT is often associated with other non-endocrine autoimmune diseases (NEAD), such as celiac diseases and chronic atrophic gastritis. Markedly higher percentages of CD24^hi^CD38^hi^ unstimulated Bregs and Th17 cells were observed in patients with HT+NEAD, but unstimulated Bregs with a memory phenotype (CD24^hi^CD38^−^ and CD24^hi^CD27^+^) were dramatically reduced. After CpG stimulation, IL-10^+^CD24^hi^CD38^hi^ Bregs were similar in patients with HT+NEAD and healthy controls (86, 87). However, it is unknown whether such changes within subsets are accompanied by functional changes. Thyroid-associated ophthalmopathy (TAO) is an autoimmune disease that threatens vision. When stimulated with CD40L and CpG, PBMCs from patients with TAO showed a decreased frequency of IL-10^+^ B cells compared to healthy controls (88). Another study conducted by the same laboratory reported that active TAO patients had higher baseline levels of IL-10^+^ B cells in their peripheral blood than inactive patients and healthy controls, though their functions were impaired (89). Above all, there are limited studies that focus on the function of Bregs in patients with AITD, functional studies will provide more information about the treatment of AITD.

### Bregs in Type 1 Diabetes

Impaired functions of IL-10^+^ B cells in type 1 diabetes (T1D) patients have been confirmed (2). The percentages of CD24^hi^CD38^hi^ B cells in PBMCs of patients with T1D were significantly lower compared to healthy controls, and these cells produced less IL-10 upon stimulation with Brefeldin A together with phorbol 12-myristate 13-acetate and ionomycin. CD24^hi^CD38^hi^ B cells in patients with T1D lacked regulatory capacity, which was related to the enhanced CD4^+^IFN-γ^+^ T cell and CD4^+^TNF-α^+^ T cell responses (2). In mice, intravenous injection of activated B cells into young NOD mice delayed disease onset and protected mice from diabetes. The therapeutic effect was related to reduced IFN-γ secretion and increased IL-4 and IL-10 production by splenocytes and T cells. However, this treatment only delayed the disease onset in old NOD mice. IL-10 was indispensable to this process because the effect disappeared when the mice were transferred with *Il-10^−/^* ^−^ B cells (90). FasL-expressing B cells were involved in the protection of diabetes by producing TGF-β, down-regulating Th1 responses and inhibiting antigen-presenting cells (APCs) to stimulate responder T cell proliferation (23). Taken together, regulatory B cells inhibit the development of T1D by reducing the pathogenic T cell-mediated tissue inflammation, then recover the function of islet β cells.

### Bregs in Multiple Sclerosis

Multiple sclerosis (MS) is characterized by chronic inflammation of the central nervous system (CNS) and axonal damage (91). Knippenberg et al. revealed that IL-10 production by Bregs from relapsing-remitting MS (RRMS) patients during relapse and RRMS patients in remission was impaired. In this study, RRMS patients in remission also had a reduced naïve (CD3^−^CD19^+^CD27^−^)/memory (CD3^−^CD19^+^CD27^+^) IL-10^+^ Bregs ratio in PBMCs (92). Partial reversal of MS was achieved with Fingolimod and Siponimod targeting sphingosine 1-phosphate (S1PR), these treatments were accompanied with increased levels of Bregs, including CD24^hi^CD38^hi^ Bregs, CD43^+^CD27^+^ Bregs, and TGF-β^+^ Bregs (93, 94). Experimental autoimmune encephalomyelitis (EAE) is one of the original models that Wolf et al. observed the suppressive effect of B cells in autoimmune models (95). Fillatreau et al. showed that toll-like receptor 9 generate proB cells (CpG-proBs) could home to reactive lymph nodes, and limited immunopathogenesis through IL-10 in EAE mice (96). Another study reported that transfusion of IL-10^+^ Bregs reversed the established clinical EAE, accompanied with CNS resident CD11b^+^CD45^int^Ly6C^−^ microglia, and infiltrating CD11b^+^CD45^high^ monocytes/macrophages content reverts to normal and polarize to a M2-like phenotype (97). IL-35-producing B cells were shown to be in the protection of EAE. Mice in which only B cells did not express IL-35 (p35 or Ebi3) lost their ability to recover from EAE (49). TGF-β1 expression in B cell was also important for the amelioration of EAE. Mice deficient for TGF-β1 expression in B cells showed an earlier onset of neurologic impairment compared to their littermate controls, associated with augmented CNS Th 1/17 responses (98). Integrin α4 was required for the immunosuppressive function of B cells in the EAE, because deletion of *Itga4* in B cells leads to EAE exacerbation and *Itga4*-sufficient B cells can control EAE severity in CD19^Cre^Itga4^fl/fl^ mice (99). Interestingly, natalizumab (targeting integrin α4) has been used to treat MS *via* inhibiting aggregation and inflammatory activity of activated immune cells, which seems to be contrary to the effect of integrin α4 on B cells in EAE (99, 100). Probably, natalizumab functions mainly through preventing the aggregation of effector cells in CNS rather than Bregs.

### Bregs in Other AIDs

**Figure 1:**
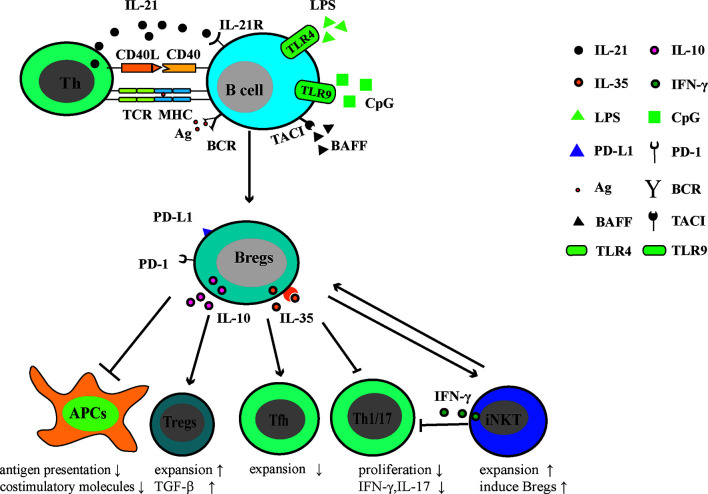
Regulatory function of Bregs in AIDs. Following exposure to the autoantigen, (presented to T cells *via* MHC molecules) CD40 signaling, Toll-like receptor (TLR) agonists (LPS or CpG), BAFF, IL-21, and/or IL-35, B cells mature into Bregs that can actively express and secrete immuno-modulatory molecules, including IL-10, IL-35, PD-1, and its ligand-PD-L1. Through these molecules, Bregs negatively regulate the antigen presentation and expression of costimulatory molecules. In Th response, Bregs restrict Tfh, Th1, and Th17 responses. In addition, Bregs promote the generation of Tregs and induce suppressive natural killer T cells. Thus, Bregs play a protective role in the development of AIDs.

Studies of Bregs in patients with other AIDs are relatively limited in comparison with SLE and RA. The frequency of CD24^hi^CD38^hi^ B cells was increased in patients with pSS, but these B cells were deficient in inhibiting IFN-γ andTNF-α production by CD4^+^ T cells (101). Tfh cells are involved in the GC formation and B cell terminal differentiation. Lin and colleagues demonstrated that Tfh cells were positively related to pSS disease severity and were negatively related to the number of IL10^+^CD24^+^CD38^hi^ B cells in pSS patients (102). Another report demonstrated the effect of Bregs on Tfh responses, in which the differentiation of CD4^+^ T_conv_ into Tfh cells was inhibited. In a Tfh-B cell co-culture system, the addition of CD40/TLR9 stimulated B cells dampened the secretion of immunoglobulins and promoted the expansion of FoxP3^+^CXCR5^+^PD-1^+^ follicular regulatory T cells (Tfr) (103). In patients with systemic sclerosis, Tim-1^+^ B cells lost the ability to inhibit autologous CD4^+^ T cell responses, including the proliferation of CD4^+^ T cells and the production of IFN-γ, TNF-α, and IL-17 (104). Patients with anti-neutrophil cytoplasmic antibodies-associated vasculitis (AAV) often have a diminished number of IL-10-producing B cells, which were correlated with the increased levels of Th1 and Th17 cells (105, 106) (**Figure 1**).

Negative relationships among disease activity, pro-inflammatory responses, and Bregs suggest that Bregs play a protective role in AIDs. Murine Bregs inhibit APCs to stimulate T cell proliferation and cytokine production (IFN-γ, TNF-α, and IL-17). Similar to murine models, human Bregs always suppress the proliferation of effector T cells and their pro-inflammatory activity. Also, human Bregs have been confirmed to indirectly dampen humoral responses *via* Tfh cells. Thus, enhancing the activity of Bregs might directly or indirectly reduce cellular/humoral responses and strengthen the function of Tregs, ameliorating inflammatory responses within AIDs.

## Potential Targets for Therapy

The majority of current treatments for AIDs are immunosuppressive drugs, such as glucocorticoids, which can result in complications in some patients during long-term use. For example, long-term prednisolone use increases the risk of osteoporosis, hypertension, and diabetes (107). Recently, cell-based therapies have been successfully used for the treatment of AIDs. These treatments include Treg-based, hematopoietic stem cell-based, and mesenchymal stem cell-based therapies (108–110).

From the above description, Bregs often play a protective role in AIDs. Therefore, it is worth considering Bregs as therapeutic targets for AIDs. Interestingly, microbiota-derived 5 Hydroxyindole-3-acetic acid (5-HIAA) suppressed murine arthritis by expanding AhR^+^IL-10^+^CD19^+^CD21^hi^CD24^hi^ Bregs, suggesting the importance of microbes in the maintenance of Bregs (111). Gut microbiota-driven IL-1β and IL-6 induced the differentiation of mouse IL-10–producing B cells with the support of CD40 signaling (12). Murine B cells stimulated with *E. coli* led to an increased production of IL-10, these B cells were capable of efficiently inhibiting the maturation and function of dendritic cells (DCs), preventing the proliferation and polarization of Th1 and Th17 cells (63). Upon exposure to inflammatory cytokines, such as BAFF, IL-1β, IL-6, IL-21, IL-33, IL-35 alone or combine with other stimuli, Bregs numerically expand or functionally enhance (12, 21, 50, 60, 112). For example, culturing B cells with BAFF can induce the differentiation of IL-10-producing B cells in mice (13). IL-35 mediated the expansion of murine Bregs that produced both IL-35 and IL-10 (50). However, the administration of cytokines may also evoke unexpected inflammatory responses *in vivo*. For example, IL-21 and CD40L could synergistically promote B cells from human tonsils into plasma cells (113), BAFF could promote B cell activation in pSS patients (114). Therefore, it might be better to transfer *ex vivo* expanded Bregs for treatment rather than administering cytokines. Mesenchymal stem cells (MSCs) are promising biological agents, and mouse CD23^+^CD43^+^ Bregs generated from B cells co-cultured with MSC for 48 h inhibited T cell proliferation *via* IL-10 (115). For some AIDs with physiologic barriers, migration is limited for circulating Bregs into inflammatory sites. Exosomes are extracellular vesicles with a size range of ~40 to 160 nm (average ~100 nm) in diameter and they can cross the physiologic barriers (116). Recently, IL-35-carrying exosomes from *ex-vivo*-generated IL-35^+^ Bregs have been reported to cross the blood-retinal barrier and suppressed experimental autoimmune uveitis *via* suppressing Th17 responses as well as inducing expansion of Tregs, with minimal toxicity and alloreactivity (117). Thus, the exosomes-mediated regulatory function of Bregs may be a promising treatment of AIDs with physiologic barriers, but it still needs further investigation. Therefore, a detailed methodology for the expansion of Bregs should be explored, which will provide better material (such as extracellular vesicles from engineered Bregs) for AIDs treatments. In this manner, the morbidity associated with AIDs will reduce and the life quality of patients will get improved.

## Conclusion

B cells are essential components of the adaptive immune system and display important roles in the pathogenesis of AIDs. With the stimulation of inflammatory cytokines and chemokines, B cells migrate into tissues and exert functions. In contrast, the identification of Bregs provides new insight into the role of B cells in AIDs. Based on the phenotypes described above, we can find that the phenotype of Bregs are varied including the phenotypes related to transitional B cells as well as highly differentiated plasma (blasts) cells. Without specific transcriptional factors or unique markers, the characterization of Bregs is usually based on their ability to secrete anti-inflammatory cytokines and express inhibitory molecules, suppressing the activity of effector cells and inducing the generation of Tregs. Considering the excessive immune responses in AIDs, Bregs act as protectors from AIDs with their immunomodulatory functions. Antigen signals (self or foreign antigens) appear to drive the development of Bregs, with many AIDs associated molecules, activators and supporters of Bregs. Thus, the adoptive transfer of *ex vivo* expanded Bregs might be a therapeutic strategy for AIDs, especially tissue-specific AIDs (such as T1D) due to the limited inflammation in the local tissues. However, there remain significant unknowns about Bregs, including specific surface markers and transcriptional factors that could be used to identify these cells, and how to maintain functional stability *in vivo*. Further investigations should focus on these unknowns, for a better understanding of Bregs and their practical application to AIDs treatments.

## Author Contributions

QZ drafted the manuscript. KR and SW discussed and revised the manuscript. JT conceived the topic and revised the manuscript. All authors contributed to the article and approved the submitted version.

## Funding

This work was supported by the National Natural Science Foundation of China (Grant Nos. 81971542, 81701612), Natural Science Foundation of Jiangsu (Grant No. BK20170563), Summit of the Six Top Talents Program of Jiangsu Province (Grant No. 2017-YY-006), the Primary Research and Development Plan of Zhenjiang (Grant SH2020041).

## Conflict of Interest

The authors declare that the research was conducted in the absence of any commercial or financial relationships that could be construed as a potential conflict of interest.
